# Hypertrophic cardiomyopathy in purpose-bred cats with the A31P mutation in cardiac myosin binding protein-C

**DOI:** 10.1038/s41598-023-36932-5

**Published:** 2023-06-26

**Authors:** Joshua A. Stern, Victor N. Rivas, Joanna L. Kaplan, Yu Ueda, Maureen S. Oldach, Eric S. Ontiveros, Kristina B. Kooiker, Sabine J. van Dijk, Samantha P. Harris

**Affiliations:** 1 Department of Medicine and Epidemiology, School of Veterinary Medicine, University of California-Davis, One Shields Avenue, Davis, CA 95616 USA; 2 Department of Clinical Sciences, College of Veterinary Medicine, North Carolina State University, 1038 William Moore Dr, Raleigh, NC 27606 USA; 3 VCA Sacramento Veterinary Referral Center, 9801 Old Winery Place, Sacramento, CA 95827 USA; 4 Rady Children’s Institute for Genomic Medicine, 7910 Frost Street, San Diego, CA 92123 USA; 5 Division of Cardiology, Department of Medicine, University of Washington, Seattle, 98109 USA; 6 Department of Neurobiology, Physiology, and Behavior, University of California-Davis, One Shields Avenue, Davis, CA 95616 USA; 7 Department of Physiology, College of Medicine-Tucson, University of Arizona, 313 Medical Research Building, 1656 E Mabel St., Tucson, AZ 85724 USA

**Keywords:** Cardiology, Cardiomyopathies, Cardiac hypertrophy

## Abstract

We sought to establish a large animal model of inherited hypertrophic cardiomyopathy (HCM) with sufficient disease severity and early penetrance for identification of novel therapeutic strategies. HCM is the most common inherited cardiac disorder affecting 1 in 250–500 people, yet few therapies for its treatment or prevention are available. A research colony of purpose-bred cats carrying the A31P mutation in *MYBPC3* was founded using sperm from a single heterozygous male cat. Cardiac function in four generations was assessed by periodic echocardiography and measurement of blood biomarkers. Results showed that HCM penetrance was age-dependent, and that penetrance occurred earlier and was more severe in successive generations, especially in homozygotes. Homozygosity was also associated with progression from preclinical to clinical disease. A31P homozygous cats represent a heritable model of HCM with early disease penetrance and a severe phenotype necessary for interventional studies aimed at altering disease progression. The occurrence of a more severe phenotype in later generations of cats, and the occasional occurrence of HCM in wildtype cats suggests the presence of at least one gene modifier or a second causal variant in this research colony that exacerbates the HCM phenotype when inherited in combination with the A31P mutation.

## Introduction

Hypertrophic cardiomyopathy (HCM) is the most common inherited cardiomyopathy in people affecting 1:250–500 individuals^1^. HCM is traditionally characterized by asymmetric or symmetric left ventricular (LV) hypertrophy with impaired diastolic relaxation. However, clinical symptoms are heterogeneous, with most patients showing no clinical signs while others develop a range of consequences including congestive heart failure (CHF), malignant arrhythmias, or sudden cardiac death^2^.

Part of the complexity in understanding preclinical and clinical HCM phenotypes is due to heterogeneity in underlying genetic causes of the disease with more than 1,000 mutations in at least 15 genes associated with HCM^1^. However, up to ~ 70% of mutations occur in two genes, *MYH7* and *MYBPC3*, encoding beta-myosin heavy chain (myosin) and cardiac myosin-binding protein-C (cMyBP-C), respectively. Myosin is the force-generating protein in muscle responsible for the power that drives cardiac contraction during each heartbeat, whereas, cMyBP-C is a regulatory protein that modifies actomyosin interactions to regulate the rate and extent of contraction and relaxation^3,4^. Because myosin, cMyBP-C, and most other proteins carrying causative mutations for HCM localize to sarcomeres (the basic contractile units of muscle), HCM has become known as a “disease of the sarcomere”. This basic understanding has led to intense efforts to target the sarcomere for drug therapies which have led to exciting new first-in-class drugs that directly affect muscle function by inhibiting or activating myosin^5,6^. While these drugs hold promise for effective treatments and potentially prevention of HCM, obstacles still remain; such as variable mutation penetrance and differences in expressivity that make it difficult to identify at-risk individuals that will benefit from treatment.

Animal models of HCM can be useful in understanding some of this variability, especially large animal (non-rodent) models that have disease etiologies and phenotypes similar to human HCM^7,8^. In this regard, HCM is the most common naturally occurring inherited cardiomyopathy in domestic cats with a reported incidence in one study as high as 15%, and is the most common cause of CHF^9^. Unlike engineered rodent models of HCM, feline HCM shows similar clinical and pathological presentations as human HCM including left ventricular outflow tract obstruction (LVOTO)^10^. Because LVOTO is a significant factor affecting patient quality of life in people^11,12^, animal models that recapitulate hemodynamic complications of HCM are especially valuable. For instance, LVOTO is not typical of genetically engineered mouse models of HCM and therefore such models are limited in the pursuit of novel therapeutics that can mitigate LVOTO gradients. HCM in cats also appears to have similar underlying genetic causes as in people; to date, two causal mutations in *MYBPC3* have been identified in Maine Coon and Ragdoll cats^13,14^. However, thus far other genetic causes have proven difficult to identify in cats and few longitudinal studies of feline HCM with a known causative mutation have been performed, thus, exacerbating gaps in our understanding of feline disease progression^15^.

Here we aimed to characterize the long-term impact on cardiac function of the A31P missense mutation in cMyBP-C first identified in Maine Coon cats^13^. The mutation results in a single amino acid substitution causing a proline to be substituted for an alanine at the protein’s 31^st^ amino acid position near the N’-terminus of the molecule (91G > C; p.A31P). Previous studies showed that the mutation disrupts the folded domain structure of the first immunoglobulin (Ig)-like domain of cMyBP-C and increases susceptibility to proteolytic degradation, however, the mutation does not lead to significant loss of cMyBP-C (haploinsufficiency)^16^. Normal levels of cMyBP-C were also reported in another cat homozygous (i.e., carrying two mutant alleles) for A31P where the mutation was reported to decrease binding to actin^17^.

Because the incidence of clinical HCM in cats heterozygous (i.e., carrying a single mutant allele) for the A31P mutation appears low in the general cat population^18,19^, we hypothesized that disease phenotype would be more penetrant in cats homozygous for the mutation. Cats were purpose bred to obtain homozygous A31P genotypes to determine whether homozygous status would constitute a useful animal model of HCM with requisite high penetrance of disease in young animals (< 1 year) ideal for preventative therapeutic studies. This study was designed to minimize bias and confounders by prospectively and blindly assessing cats housed in a controlled environment over time from kitten to adult ages. Cats homozygous for the A31P mutation develop preclinical asymmetric ventricular growth and diastolic dysfunction suggesting that continued selective breeding in this unique research colony will be valuable for identifying interventional therapies for both human and veterinary medicine.

## Results

### Colony breeding and pedigree relationships

**Figure 1:**
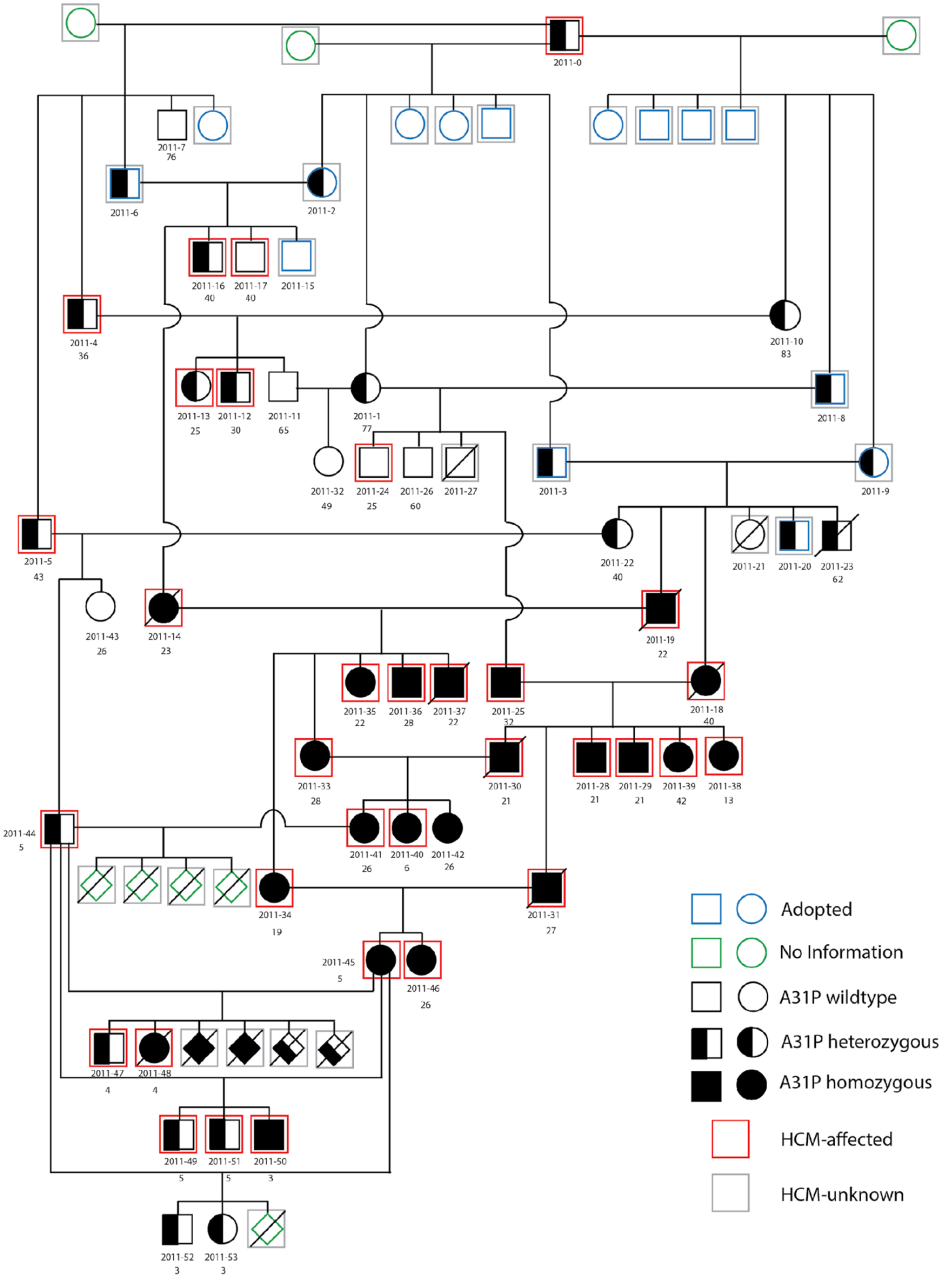
Extended A31P cat colony pedigree. Pedigree representing a family of 74 cats (35 males, 30 females, and 9 cats of unknown sex) derived from single male founder cat heterozygous for the A31P mutation. Squares = males, circles = females, and diamonds = kittens of unknown sex that were either stillborn or died secondary to failure to thrive. Open symbols are wildtype cats, half-filled symbols are heterozygous A31P cats, and filled symbols are homozygous A31P cats. Green symbols are cats of unknown genotype. Forty-four cats were phenotyped via echocardiography for HCM. Red squares indicate HCM-affected cats; HCM affection status was strictly defined as the following: (1) a diastolic LV wall segment ≥ 6 mm identified in at least two separate echocardiographic examinations in the absence of any other systemic or metabolic disease, or (2) a diastolic LV wall segment ≥ 6 mm identified on a single echocardiographic examination in conjunction with a serum NT-proBNP concentration of > 100 pmol/L^20^ in the absence of any other systemic or metabolic disease. Gray squares indicate cats that were not phenotyped or that have an unknown affected status. Blue symbols are cats that were adopted and were not included in longitudinal phenotyping analyses. Cats without a red or gray square represent those with a confirmed HCM-unaffected phenotype at the time of last echocardiographic evaluation. The pedigree chart is labeled with the unique identification number for each cat (#2011-XX). Age in months at time of first echocardiographic evidence of HCM are listed below unique cat identification numbers. Age (months) at time of last echocardiographic evaluation is reported for cats that did not meet HCM affection status below unique cat identification numbers. HCM: hypertrophic cardiomyopathy; LV: left ventricle; NTproBNP: N-terminal pro-brain natriuretic peptide.

The breeding strategy used to obtain cats carrying the A31P mutation in cMyBP-C is presented in Fig. 1. The A31P mutation in exon 3 of *MYBPC3* was first identified in a research colony of cats with HCM and was also identified in Maine Coon cats in general cat populations^13^. However, the original research colony consisted of a mixed genetic population of cats with HCM and not all cats in that colony with HCM carried the A31P mutation. Therefore, multiple genetic etiologies likely contributed to HCM in the original colony^20^. To focus exclusively on disease progression from a known genetic cause, we selected a single male cat from the original HCM colony that was heterozygous for the A31P mutation. Sperm from this heterozygous A31P male was used to develop a second-generation research colony using artificial insemination of three healthy, unrelated, wildtype DSH (domestic shorthair) female cats. Resulting heterozygous A31P cats were crossed to obtain cats homozygous for the A31P mutation. Homozygous A31P cats were born in Mendelian ratios and survived into adulthood. Homozygous X homozygous crosses produced healthy litters, albeit, litter sizes were frequently small for all colony breeding pairs regardless of genotype (typically two-to-five kittens).

### Longitudinal echocardiographic assessments

Cardiac structure and function was assessed by echocardiography in kittens three-to-six months of age and thereafter at regular intervals of six-to-twelve months throughout the term of the study. Standard cardiac morphology and functional indices were measured via M-mode, 2-D, and color Doppler imaging for a complete echocardiographic exam at each time point. Blood samples were also collected at each exam for analysis of NT-proBNP and cardiac troponin-I (cTnI) serum biomarkers. Affected status was determined after each exam according to criteria described in “Methods”.

**Figure 2:**
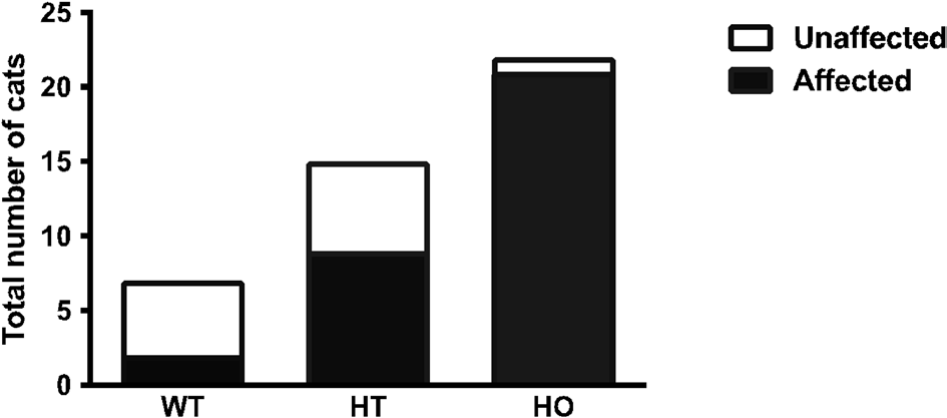
Proportion of HCM-affected status by genotype. The proportion of unaffected and affected cats for each genotype is shown. Of the seven wildtype (WT) cats, five were unaffected and two were affected. Fifteen were heterozygous (HT) for the A31P variant, of which six were unaffected and nine were affected. Of the 22 cats homozygous (HO) for the A31P variant, one cat was unaffected and 21 were affected. HCM: hypertrophic cardiomyopathy; WT: wildtype, HT: heterozygous, HO: homozygous.

Analyses of longitudinal echocardiographic assessments revealed gradual progression of preclinical disease penetrance in most colony cats. The progression was evident as an increase in cats designated affected with increasing number of evaluations, increasing age at time of exam, and with A31P homozygous genotype (Fig. 1). As expected, penetrance was greatest in cats homozygous for the A31P mutation and was least for wildtype cats without the mutation; however, some wildtype cats without the A31P genotype also progressed to affected status (Fig. 2). Individual cardiac indices were next grouped and analyzed according to A31P genotype. Statistically significant differences were achieved for final measurements of interventricular septal (IVS) diameter in diastole, and absolute left atrial (LA), and normalized LA-to-aortic root (LA/Ao) dimensions between wildtype cats versus cats homozygous for the A31P mutation (Table 1). To ensure that compensatory effects of cardiac remodeling did not blunt detection of greater differences at earlier time points, we also compared highest (or lowest) individual values obtained for each index regardless of final value recorded at the last time point. Statistically significant differences were found for Ao and LA/Ao values between genotypes (Table S1).

**Table 1:** Median (IQR) values of echocardiographic parameters obtained during final echocardiographic examinations by A31P genotype.

Parameter	Genotype			P-value			
	HO (n = 23)	HT (n = 14)	WT (n = 7)	Overall	HO vs. HT	HO vs. WT	HT vs. WT
IVS diastole (cm)	0.58 (0.48–0.62)	0.51 (0.49–0.61)	0.47 (0.35–0.53)	0.047*	> 0.9999	0.04*	0.25
IVS systole (cm)	0.91 (0.77–0.98)	0.84 (0.77–1.02)	0.81 (0.65–0.95)	0.34			
LV diastole (cm)	1.21 (1–1.38)	1.29 (1.09–1.5)	1.14 (1.07–0.32)	0.29			
LV systole (cm)	0.46 (0.33–0.56)	0.54 (0.42–0.73)	0.51 (0.43–0.67)	0.25			
LVPW diastole (cm)	0.57 (0.49–0.63)	0.52 (0.49–0.56)	0.46 (0.34–0.58)	0.09			
LVPW systole (cm)	0.83 (0.77–1)	0.86 (0.73–0.96)	0.78 (0.58–0.88)	0.43			
FS (%)	66.05 (57.–70.88)	56.4 (51.2–65.4)	52.4 (47.7–65.4)	0.056			
LA diastole (cm)	1.29 (1.23–1.41)	1.26 (1.15–1.39)	1.16 (1.02–1.29)	0.041*	> 0.9999	0.035*	0.24
Ao systole (cm)	0.84 (0.8–0.87)	0.91 (0.85–0.97)	0.93 (0.86–0.94)	0.031*	0.1	0.097	> 0.9999
LA/Ao	1.57 (1.52–1.61)	1.42 (1.35–1.46)	1.23 (1.22–1.25)	< 0.0001*	0.0083*	< 0.0001*	0.19
E Vmax (m/sec)	0.68 (0.49–0.87)	0.62 (0.56–0.69)	0.62 (0.50–0.72)	0.67			
A Vmax (m/sec)	0.79 (0.65–0.85)	0.73 (0.61–0.82)	0.78 (0.66–1.01)	0.59			
E/A (m/sec)	0.74 (0.62–0.83)	0.8 (0.69–1.17)	0.63 (0.57–0.77)	0.13			
LAA flow (m/sec)	42.8 (34.76–48.84)	41.91 (37.39–48.3)	48.97 (41.03–59.11)	0.13			
Septal bulge (mm)	6.3 (6–7.2)	6.15 (5.5–6.7)	5.6 (5.4–6.33)	0.1			
Ao Vmax (m/sec)	1.45 (0.98–2.24)	1.82 (0.92–2.24)	1.03 (0.75–3.28)	0.73			
P Vmax (m/sec)	1.14 (0.96–1.37)	0.97 (0.79–1.53)	1.84 (0.74–2.22)	0.35			

Ao: aorta, Ao V_max_: peak velocity of aortic flow, A V_max_: peak velocity of late transmitral flow, E V_max_: peak velocity of early diastolic transmitral flow, FS: fractional shortening, IVS: interventricular septum, LAA: left auricular appendage, LV: left ventricle, LVPW: left ventricular posterior wall, P V_max_: peak velocity of pulmonary flow, HT: heterozygous, HO: homozygous, and WT: wildtype. *P < 0.05.

LVOTOs were also common (present in ~ 18% of examinations) and dynamic within the colony. Although dynamic LVOTO was occasionally observed in wildtype cats, the incidence of mid-ventricular obstruction was common in cats with the A31P variant and HCM affected status. Juvenile cats with early onset and severely affected disease status also commonly demonstrated LVOTO. As seen in Table S1, cats that were heterozygous or homozygous for the A31P mutation had median aortic flow velocities at or above 2 m/sec, constituting LVOTO in these genotype groups compared to wildtype cats with a median of 1.4 m/sec^21–23,55^.

**Figure 3:**
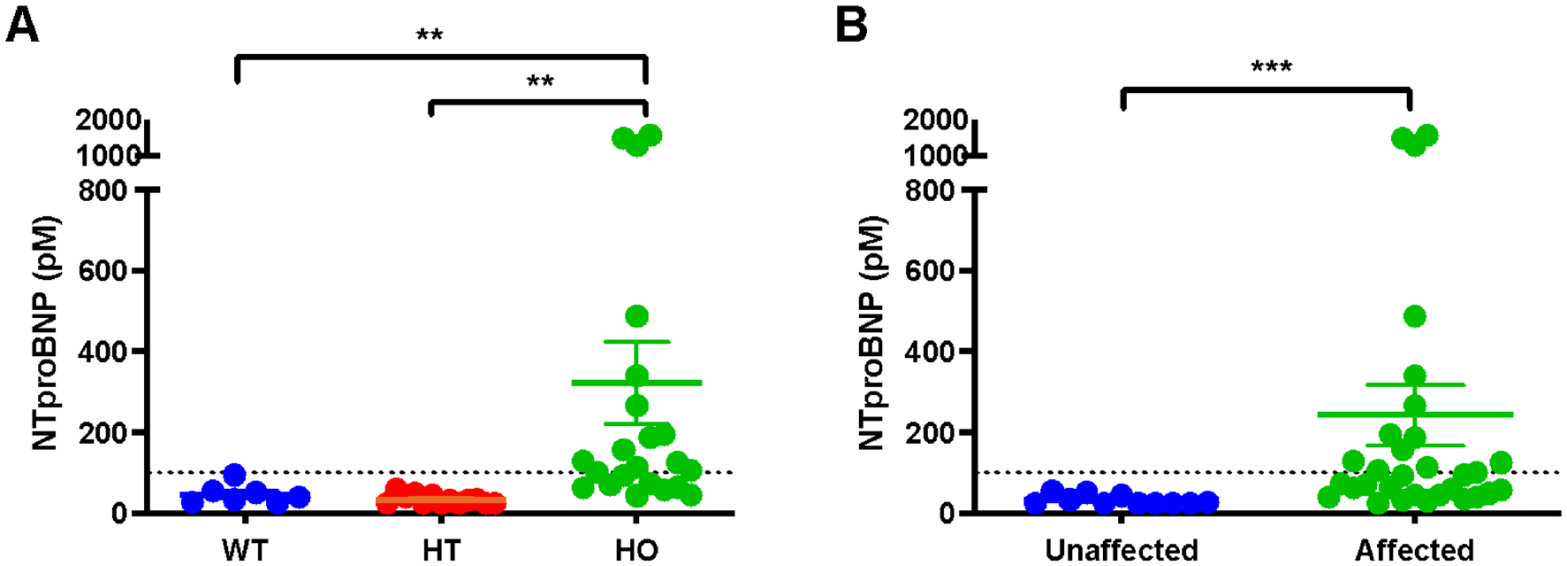
Plasma NT-proBNP values in A31P cat colony. (**A**) Highest single measurement of plasma NT-proBNP concentration by genotype. (**B**) Highest measured plasma NT-proBNP concentration in HCM-unaffected and -affected cats. Dotted line represents 100 pM, the clinically accepted cut off value for high NT-proBNP concentration. ** *P* < 0.008, *** *P* < 0.0001. NTproBNP: N-terminal pro-brain natriuretic peptide; HCM: hypertrophic cardiomyopathy.

NT-proBNP values were significantly associated with HCM affected status, A31P genotype, and the presence of LVOTO in colony cats (Tables 2 and 3). This is consistent with the observed enlargement of the LA and LV thickening that predominates in HCM-affected A31P homozygous cats within this study. Increased serum NT-proBNP values were significantly associated within affected cats, specifically homozygotes when compared to other genotypes (Fig. 3). Serum cTnI values were not significantly different between affected and unaffected cats (data not shown).

**Table 2:** Highest plasma NT-proBNP concentrations were compared based on sex, age, body weight, HCM status, A31P genotype, and the presence or absence of LVOTO.

NT-proBNP value	P-value	R
HCM	0.003*	
A31P genotype	< 0.0001*	
LVOTO	0.023*	
Sex	0.23	
Age	0.62	− 0.078
Body weight	0.94	0.011

Correlation analyses were performed between the highest NT-proBNP concentrations and continuous variables including age and weight. Significant correlations between NT-proBNP concentrations and HCM affected status, A31P genotype, and LVOTO status were assessed by multiple regression analysis. *P < 0.05.

HCM: hypertrophic cardiomyopathy, LVOTO: left ventricular outflow tract obstruction, NT-proBNP: N-terminal pro-brain natriuretic peptide.

**Table 3:** Multiple regression analysis on HCM status, genotype, and left ventricular outflow tract obstruction.

NT-proBNP value	Coefficient	Std. Err	P-value	95% CI		R^2^	Adjusted R^2^
HCM	49.69	125.37	0.69	− 204.11	303.49		
A31P genotype	128.26	75.24	0.096	− 24.07	280.58		
LVOTO	203.03	115.99	0.088	− 31.79	437.84		
Overall			0.026*			0.21	0.15

Multiple regression analysis was performed with backward and forward selection using *P* < 0.15.

HCM: hypertrophic cardiomyopathy, LVOTO: left ventricular outflow tract obstruction, and CI: confidence interval*,* NT-proBNP: N-terminal pro-brain natriuretic peptide.

Inheritance of the A31P mutation in homozygous cats leads to a statistically significant steady progression of preclinical indices of HCM (e.g., regional wall thickening and LA/Ao ratios); homozygotes are more likely to develop HCM compared to heterozygotes (OR = 8.5 and OR = 3.75, respectively) (Table 4). However, the odds ratio for heterozygous A31P status did not achieve statistical significance. Multiple logistic regression analysis was subsequently performed, and the homozygous A31P genotype remained significantly associated with the development of HCM (OR = 11.8) (Table S2).

**Table 4:** Odds ratios of HCM in A31P cat colony.

HCM status	Odds ratio	95% CI	P-value
A31P genotype: HO (vs. WT)	8.5	1.25–57.93	0.029*
A31P genotype: HT (vs. WT)	3.75	0.54–26.04	0.18
Body Weight	1.48	0.93–2.38	0.1
NT-proBNP	1.01	1.0–1.03	0.094
Age	1.01	0.97–1.05	0.66
Sex: female (vs. male)	0.93	0.26–3.31	0.91

Odds ratios were calculated using logistic regression analysis between HCM status and A31P genotypes, age, sex, and body weight. *P < 0.05.

WT: wildtype, HT: heterozygous, HO: homozygous, NT-proBNP: N-terminal pro-brain natriuretic peptide, and CI: confidence interval.

### Preclinical to clinical HCM progression of A31P homozygotes

**Figure 4:**
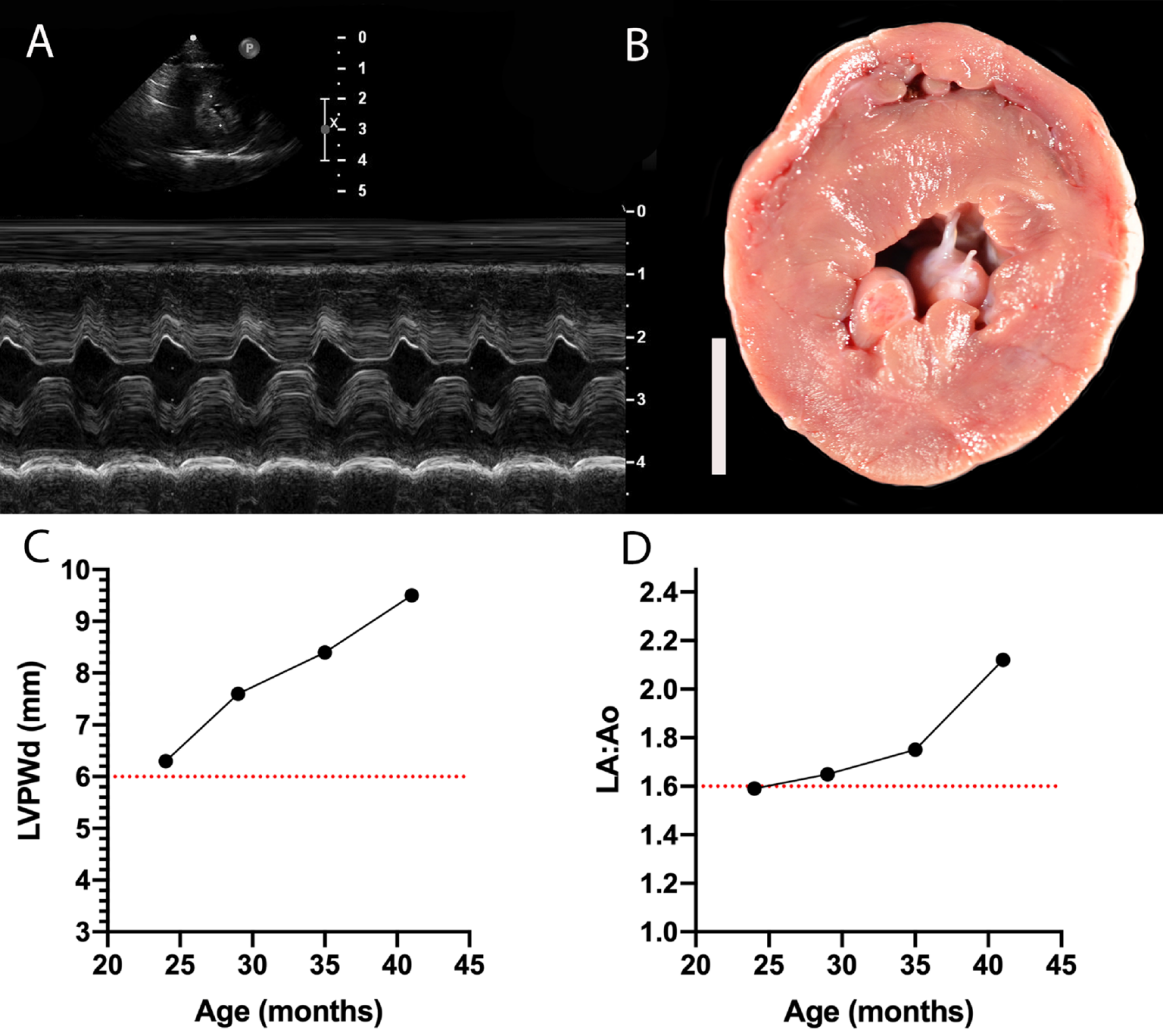
Echocardiography and gross pathology of a severe HCM-affected cat homozygous for the *MYBPC3* A31P mutation (cat #2011-14). (**A**) M-mode image of the LV taken from the right parasternal short axis imaging plane documenting severe LV hypertrophy in a female cat at 3.5 years of age. (**B**) A coin-cut gross pathology image of LV showing severe wall thickening and prominent papillary muscles; white bar = 1 cm. (**C**) Longitudinal echocardiographic data of the LVPWd from the right parasternal short axis imaging plane over sequential evaluations. (**D**) Longitudinal echocardiographic data of LA/Ao in 2D from the right parasternal short axis imaging plane over sequential evaluations. The final evaluation in C and D was completed at ~ 3.5 years of age, immediately prior to euthanasia for new onset left-sided congestive heart failure. Dotted red lines (**C**) & (**D**) represent the threshold for LVPWd measures consistent with HCM and LA/Ao consistent with LA enlargement. HCM: hypertrophic cardiomyopathy; MYBPC3: myosin-binding protein-C; LV: left ventricle; LVPWd: left ventricle posterior wall in diastole; LA/Ao: left atrium to aortic root ratio; LA: left atrium.

Disease penetrance, progression, and severity in cats homozygous for the A31P mutation have not been well described due in part to the small number of homozygous cats available for study in general cat populations. Here we studied 22 cats homozygous for the A31P mutation. Of these, three cats progressed from preclinical status to CHF during the course of the study. The first cat that progressed from preclinical to clinical disease was a cat from the first generation of cats obtained from heterozygous X heterozygous crosses (cat #2011-14, Fig. 1). Compared to other homozygous cats within the same generation, cat #2011-14 had more severe, uniform, and rapidly progressive LV hypertrophy (Fig. 4). Cat #2011-14 had abnormal LV diastolic wall thickness at 23 months of age and increased LA size at 29 months of age that increased to severe enlargement by the final echocardiographic evaluation. CHF occurred at 41 months as evidenced by severe LA enlargement, pulmonary edema, and pericardial effusion.

### A31P allelic interactions and association with early onset, severe, HCM phenotypes

**Figure 5:**
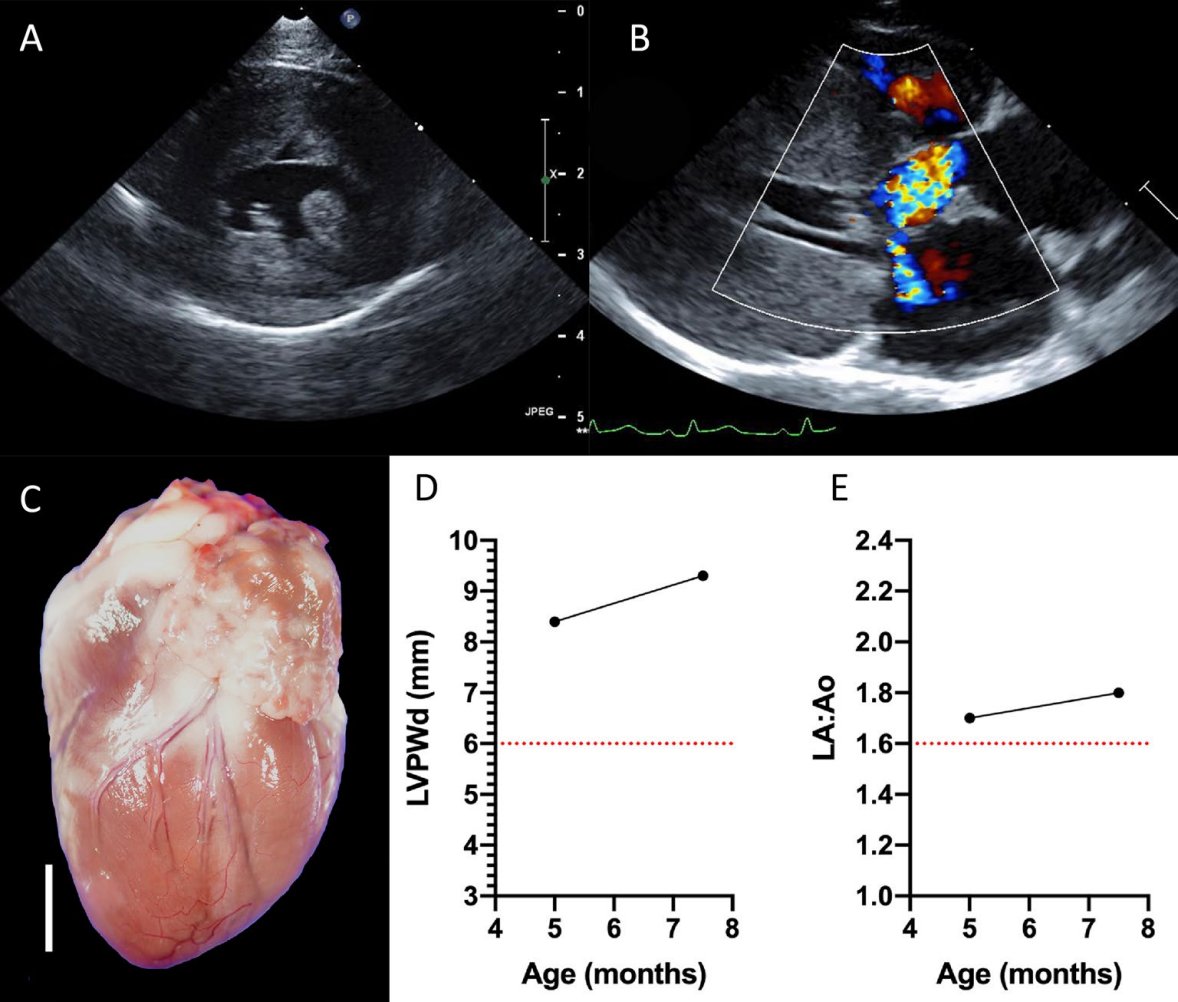
Echocardiography and gross pathology of a severe, early onset, HCM-affected female cat (cat #2011-48) homozygous for the *MYBPC3* A31P mutation. (**A**) 2D echocardiographic image of the LV from the right parasternal short axis imaging plane documenting severe LV hypertrophy at ~ 5 months of age. (**B**) 2D and color Doppler echocardiographic image of the LVOT from the right parasternal long-axis imaging plane showing turbulence in the LVOT secondary to SAM and associated mild mitral valve regurgitation. Severe LV hypertrophy is again shown in this imaging plane. (C) Gross pathology image of the intact heart showing a prominent LV and dilated LA and LA appendage; white bar = 1 cm. (**D**) Longitudinal echocardiographic data of the LVPWd from the right parasternal short axis imaging plane over 2 sequential evaluations. (**E**) Longitudinal echocardiographic data of LA/Ao in 2D from the right parasternal short axis imaging plane over sequential evaluations. The final evaluation in (**D**) and (**E**) was completed at ~ 7.5 months of age, just prior to euthanasia for new onset left-sided congestive heart failure. Dotted red lines (**D** & **E**) represent the threshold for LVPWd measures consistent with HCM and LA/Ao consistent with LA enlargement. HCM: hypertrophic cardiomyopathy; MYBPC3: myosin-binding protein-C; LV: left ventricle; LVPWd: left ventricle posterior wall in diastole; LA/Ao: left atrium to aortic root ratio; LA: left atrium; LVOT: left ventricular outflow tract; SAM: systolic anterior motion of the mitral valve.

In subsequent generations, HCM affected status occurred at earlier times either at first echocardiographic exam (typically between three-to-six months of age) or before one year-of-age. Individuals that resulted from a cross involving a heterozygous male (#2011-44) and homozygous female (#2011-45) cat presented with a more severe phenotype at earlier ages than in previous generations. For instance, the second cat that progressed to CHF occurred in later generations (cat #2011-48, Fig. 1). The initial echocardiographic exam for this cat at four months showed severe diastolic IVS and LV posterior wall (LVPW) thickness of 9 and 8 mm, respectively, and mild LA enlargement (Fig. 5). HCM progressed rapidly to CHF seen as pulmonary edema, and scant pleural and pericardial effusions at 10 months of age. A third homozygous cat that developed CHF by 30 months of age was also produced from this same breeding pair (cat #2011-50, Fig. 1).

### DNA sequencing and variant analysis

Because of the relatively high degree of inbreeding in the closed colony until this point (Fig. 1), we hypothesized that the appearance of the more severe phenotype may be due to co-inheritance of a modifier variant or an independent HCM variant introduced by the male founder or by one of the three unrelated female founder cats. We therefore used whole-genome sequencing (WGS) to identify sequence variants in 15 colony cats, resulting in a total of 89,817,458 variants identified. After quality control and elimination of variants in intergenic regions, 32,218,731 variants remained. A total of 380,695 variants were localized to genes previously or suspected to be associated with HCM or in electrophysiology genes (Table S3). Of these, 15 showed statistically significant association with disease phenotype via Chi-square testing. To determine the frequency of these variants in the general cat population, we next calculated the allele frequency of the 15 variants in 195 unrelated, unphenotyped cats from a feline genetics consortium (99Lives Cat Genome Sequencing Initiative; http://felinegenetics.missouri.edu/99lives/scientist-information). Only the previously identified *MYBPC3* A31P variant was identified in the majority of HCM affected colony cats and not in any of the wildtype control cats or the general cat population (Table S4).

## Discussion

This study describes a comprehensive longitudinal assessment of HCM progression in a purpose-bred research colony of cats carrying a single known deleterious missense mutation in *MYBPC3,* the gene encoding the cMyBP-C protein. The mutation results in a proline for alanine substitution at codon 31 (A31P) and was first identified in an inbred research HCM cat colony^13^. This mutation was also found in general populations of client-owned cats of the Maine Coon breed, making it the first identified, naturally occurring, causal mutation for HCM in any companion animal species^24,25^. Because heterozygous inheritance of the A31P allele in general cat populations was associated with low disease penetrance^18,19,26^, we bred cats to achieve homozygosity for the A31P allele. Our goals were to determine 1) whether homozygous inheritance of the A31P mutation was associated with higher disease penetrance and a more severe phenotype as a prerequisite for use of A31P cats as a feline model of HCM, and 2) provide a controlled, longitudinal assessment of preclinical disease progression in a population of purpose-bred cats with and without the A31P mutation. Result from this study confirm that the A31P mutation is causative for HCM, especially when inherited in the homozygous state (OR = 8.5, Table 4). We conclude that purpose-bred cats homozygous for the A31P cMyBP-C mutation provide a reliable model of inherited HCM.

Significant findings by echocardiography included characteristics commonly reported for preclinical disease in both people and cats including regional and/or global LV wall thickening, increased diameter of the LA, and increased LA/Ao dimensions in HCM-affected cats. Additional findings included frequent indicators of diastolic dysfunction as assessed by Doppler imaging. Consistent with diastolic dysfunction, NT-proBNP concentrations were also elevated in A31P cats as previously reported^27^. NT-proBNP concentrations were highest in homozygotes and were also frequently elevated in HCM-affected cats. However, not all severely affected cats had elevated NT-proBNP. For instance, cat #2011-44 (Fig. 1) was severely affected, but NT-proBNP concentrations measured in repeated blood samples consistently yielded values in the normal range. Singh et al^28^ also reported that NT-proBNP concentration did not correlate with preclinical disease and that some severely affected Maine coon cats did not show elevated NT-proBNP. Biologic variability in NT-proBNP has also been reported in healthy cats^29^. Because NT-proBNP concentrations were also lower in a subgroup of sarcomeric mutation negative HCM patients^30^, low NT-proBNP concentration alone may not exclude disease.

Systolic abnormalities included dynamic LVOTO and systolic anterior motion of the mitral valve (SAM) frequently observed in affected cats. Consistent with previous reports, LVOTO was not associated with higher incidence of adverse outcomes^31^, although the relatively young ages and the small number of cats here precludes definitive conclusions regarding outcome correlations with LVOTO. All other parameters, including systolic function assessed by fractional shortening (FS) and ejection fraction (EF), were not significantly different in affected cats.

Taken together, these echocardiography results establish that cats homozygous for the A31P mutation reliably develop preclinical HCM with characteristics common to both human and feline diseases. Purpose-bred cats homozygous for the A31P mutation therefore represent a unique, naturally occurring, large animal model of HCM that can offer significant advantages over existing engineered rodent models of disease. For instance, while genetically engineered rodent models of HCM are essential for confirming deleterious effects of mutations identified in people^32,33^, mouse models cannot recapitulate significant aspects of disease comorbidities including hemodynamic complications such as LVOTO or susceptibility to arrhythmia. Because LVOTO is a significant factor impacting human patient quality of life^11,12^, non-rodent models are critically needed to identify new therapeutics that reduce LVOTO gradients.

Another advantage of homozygous A31P cats as an animal model of disease is that HCM occurs naturally as a result of an inherited mutation so that additional surgical, drug, or dietary interventions are not required to induce cardiac dysfunction as in other models of cardiac dysfunction^34–37^. This facilitates wider implementation in laboratory or clinical settings where access to secondary interventions may be limited. Because mutations in *MYBPC3* are the most common cause of HCM in people, understanding the natural history of HCM due to a cMyBP-C mutation in cats may offer insights into critical points of decompensation as well as provide a highly relevant model for testing new drug or gene therapies^37–39^.

Most missense *MYBPC3* mutations identified in people lead to premature protein truncation^40^. However, missense mutations coding for single amino acid substitutions, like A31P, also occur in people. In this respect, the A31P mutation occurs in the cardiac-specific C0 domain of cMyBP-C and other missense mutations have been identified in the C0 domain of HCM patients^41^. The precise function of C0 is not yet known, but C0 binds to both F-actin and the regulatory light chain (RLC) of myosin^42,43^. Disruption of these interactions could therefore affect the ability of cMyBP-C to activate the thin filament or to stabilize the super-relaxed (SRX) state of myosin^44–46^, either of which would be expected to directly cause contraction or relaxation abnormalities resulting in a cardiomyopathy.

While a second missense mutation in *MYBPC3* (encoding R820W) was identified in Ragdolls^14^, cats expressing the R820W mutation are not currently available for research studies. HCM and dilated cardiomyopathy (DCM) have been identified as naturally occurring diseases in a number of large animals including dogs, pigs, and non-human primates, but a genetic basis has yet to be established in these species. Purpose-bred cats expressing the A31P mutation in cMyBP-C represent a unique genetic model of HCM with relevance to human disease and are currently the only available naturally occurring HCM model with a defined genetic mutation.

Despite significant advantages offered by use of purpose-bred A31P cats, challenges still remain in their development as an effective model of HCM. First, HCM penetrance in cats and people is variable with different expressivity observed even in closely related individuals that carry the same mutation. Here we attempted to overcome this limitation by breeding cats to homozygosity as gene dosage effects can lead to more severe disease phenotypes. This strategy led to higher penetrance of affected status in cats with a homozygous genotype relative to heterozygous cats carrying a single copy of the mutant allele or to wildtype cats that did not express the mutation (Fig. 1). However, disease severity and time to affected status were still variable in cats homozygous for the A31P mutation, especially in the first generations of cats bred to homozygosity.

One explanation for the observation that affected status occurred earlier with successive generations of breeding is that inbreeding within the closed colony led to co-selection of another heritable modifier or causative variant resulting in an earlier, more severe phenotype when combined with the A31P mutation. A second variant could have been inadvertently introduced into the colony via one of the three apparently healthy female cats given the high incidence of asymptomatic HCM-affected individuals within client-owned populations^9,31^. Alternatively, a second variant could have been introduced by sperm from the founder male as this male was acquired from a research colony of cats bred for HCM with or without the A31P mutation^20^. However, it seems unlikely that inheritance of an unidentified variant alone can account for HCM in this colony, because WGS sequence analysis identified only the A31P mutation in the majority of colony cats with HCM (Table S4). Continued selective breeding of cats with the most severe, early, phenotypes should result in continued improvement of model traits and provide further opportunities to identify disease modifiers or other variants through sequencing of severely affected cats. The role of environmental and genetic modifiers is increasingly acknowledged as an underlying cause of disease heterogeneity in HCM patients^47–51^. Combined use of A31P cats with other interventions designed to induce cardiac dysfunction^34,35,52^ may also result in a more severe phenotype that progress to CHF over shorter, more defined, time frames.

There are several limitations to this study. First, it is well known that there is considerable variability in HCM disease penetrance even in closely related individuals that carry the same genetic mutation^53^, implying that there are significant other genetic or environmental factors that contribute to disease etiologies. Here we attempted to minimize the impact of environmental factors and genetic factors that could account for the differences in disease penetrance by maintaining a closed breeding colony with a single known genetic mutation linked to disease. However, we still observed considerable difference in disease penetrance that could not be accounted for by environment or identified genetic factors. An additional limitation of our longitudinal study design is that older generations of cats received more screening examinations than the youngest generation and thus not all cats were followed for the same amount of time. For echocardiography, cats were sedated and so potential effects of sedation on echocardiographic results cannot be excluded. Also, blood pressure measurements were not routinely performed. However, all cats with a diagnosis of HCM had a blood pressure recorded to be < 160 mmHg by Doppler Sphygmomanometry in combination with normal renal function as assessed by serum creatinine concentration within the normal reference range. Finally, histopathology analyses were not performed.

In conclusion, we describe a second-generation research colony of purpose-bred cats selected to carry a single genetic mutation in *MYBPC3.* Results from this study confirm deleterious effects of the A31P mutation in cMyBP-C by showing that cats homozygous for the mutation reliably develop preclinical indicators of HCM at early time points often before the first year of age. Cats carrying the A31P mutation should therefore be valuable for interventional studies designed to reverse or slow progression of HCM using novel and next-in-class therapeutic approaches.

## Methods

### Colony breeding and periodic assessments

A purpose-bred colony of cats was created for research studies through selective breeding of cats with the A31P mutation in cMyBP-C^13^. All aspects of animal care and use, including ethical usage of animals in research, were approved and performed in accordance with the Institutional Animal Care and Use Committee (IACUC) of the University of California Davis (protocol #22,376). In addition, this study was carried out in compliance with the ARRIVE guidelines (http://www.nc3rs.org.uk/page.asp?id=1357), the Animal Welfare Act, and the Institute for Laboratory Animal Research Guide for the Care and Use of Laboratory Animals. Priority at all times was given to cat comfort, health, and well-being. Briefly, cats were housed together in social groups in large enclosures with access to natural light, food and water (ad libitum), environmental enrichment including toys, scratching posts, perches, and beds, and were provided daily socialization with caretakers. Importantly, cats in the colony are maintained in a pre-clinical state, meaning they are without signs or symptoms of HCM and so experience minimal, if any, discomfort. Upon first development of CHF symptoms, cats are humanely euthanized.

The colony was founded using sperm from a single male Maine Coon/mix breed cat that was heterozygous for the A31P mutation obtained from a previously established research colony of cats with multiple causes of HCM at UC Davis^13,20^. Sperm from the founder cat was used to artificially inseminate three unrelated domestic short hair (DSH) females. Heterozygous A31P cats were then bred to obtain cats homozygous for the A31P mutation. A full colony pedigree chart is shown in Fig. 1 for the study period of ~ seven years (circa 2011–2018). Note that due to the finite study design, older generations of cats received more follow up screening than cats in younger generations.

All cats were genotyped and underwent periodic complete echocardiography and cardiac biomarker assessment (details provided in “Echocardiography” and “Biomarker measurements”). Examinations occurred approximately every six-to-twelve months with initial evaluation occurring between three-to-six months of age. Cardiovascular examinations were performed under sedation with a combination of butorphanol (0.2–0.3 mg/kg intramuscularly or intravenously) and acepromazine (0.05–0.1 mg/kg intramuscularly or intravenously) as needed to facilitate evaluation with minimal manual restraint.

HCM affected status was strictly defined by one of two possible scenarios: 1) a diastolic LV wall segment ≥ 6 mm identified in at least two separate echocardiographic examinations in the absence of any other systemic or metabolic disease, or 2) a diastolic LV wall segment ≥ 6 mm identified on a single echocardiographic examination in conjunction with a serum NT-proBNP concentration of > 100 pmol/L^54^ in the absence of any other systemic or metabolic disease. LVOTO was defined as color flow turbulence in association with a region of obstruction (LVOTO secondary to SAM or mid-ventricular) and a measured flow velocity of ≥ 2 m/sec, as previously reported^21–23,55^. All cats diagnosed with HCM were determined to have normal renal function via biochemistry, absence of any other known metabolic disease, a systolic blood pressure < 160 mmHg via Doppler sphymomanometry, and a total thyroxine level within the reference range if over the age of six years. CHF was defined as the presence of pleural and/or pericardial effusion via thoracic ultrasound and/or pulmonary edema on thoracic radiographs in conjunction with clinical signs of tachypnea or dyspnea. Cats diagnosed with CHF were euthanized at the first signs of clinical progression and no cats were maintained beyond diagnosis of CHF to ensure that there were no opportunities for cats to go untreated with progressive respiratory distress.

### Genotyping

DNA for PCR genotyping was extracted from either buccal swabs or EDTA blood samples for all cats. Genotyping for the A31P mutation was carried out either by the UC Davis Veterinary Genetics Laboratory or by standard PCR and Sanger sequencing as previously reported^56^.

### Echocardiography

Echocardiographic examinations were performed by a single, board-certified, veterinary cardiologist (J.A.S) using a 12-4 mHz sector-array transducer (Philips iE33 Ultrasound, Philips Healthcare, Andover, MA) and a standard approach^55^ of two-dimensional, M-mode, color, and spectral Doppler modalities. Cats were monitored via a continuous lead II ECG throughout the echocardiogram when tolerated at each evaluation. Sedated cats were first manually restrained in right then left lateral recumbencies to obtain echocardiographic images. All images were obtained and stored offline for measurement and analysis by a single, board-certified, veterinary cardiologist (J.A.S) using commercially available software and workstation (Syngo Dynamics, Siemens Medical Solutions, Malvern, PA). Cat images were identified by six-digit numbers such that the investigator was blinded to cat age, gender, and A31P genotype during measurement. When possible, the average value of three consecutive cardiac cycles was obtained for each measurement for all two-dimensional or M-mode measures. Spectral Doppler measures were recorded as the peak modal velocity. Measurements were taken to avoid ectopic cardiac cycles or cycles immediately following ectopy. LV wall measurements were obtained from both 2D and M-mode imaging as previously described with care to avoid the insertion sites of observed moderator bands^9^. LA diameter and LA/Ao measured by 2D echocardiogram in right parasternal short axis was obtained as previously described^57^. The left atrium was considered enlarged if the maximal left atrial diameter was ≥ 16 mm or the left atrium to aortic root ratio (LA:Ao) was ≥ 1.6^58,59^. Peak LV outflow tract velocity was obtained from the left apical imaging plane with parallel alignment to blood flow and noted turbulence by color-flow Doppler imaging.

### Biomarker measurements

Cardiac biomarkers were assessed in venous blood samples obtained from each cat at each cardiovascular examination when possible. NT-proBNP (Cardiopet® proBNP) and high-sensitivity cardiac troponin-I (Hs-cTnI, Beckman Access AccuTnI test) were measured in serum by a diagnostic laboratory according to sample handling and preparation guidelines (IDEXX Research & Development, IDEXX Laboratories Inc, Westbrook, ME). All cats had an indirect ophthalmic examination performed during one of their final two examinations to rule out retinal lesions consistent with systemic hypertension.

### Statistical evaluation

Statistical analyses were performed using commercially available software packages (Prism v8.2.1, GraphPad Software, La Jolla, CA; STATA v15, Stata Corporation, College Station, TX). Normality testing for continuous echocardiographic and patient characteristic variables was done using D’Agostino-Pearson test. Descriptive statistics were performed and reported as mean ± SD for parametric values and median and interquartile range (IQR) for nonparametric values.

Echocardiographic parameters obtained during the last examination were compared among cats with different A31P genotypes using Kruskal–Wallis test with Dunn’s post-hoc comparison. The highest (IVS diastole, IVS systole, LVPW diastole, LVPW systole, LA diastole, Ao systole, LA/Ao, A V_max_, Septal bulge, AoV, and PV) or lowest values (LV diastole, LV systole, FS, E V_max_, E/A, and Lar Flow) of echocardiographic parameters were also compared among cats with different A31P genotypes using Kruskal–Wallis test with Dunn’s post-hoc comparison.

The highest NT-proBNP values in the repeated measurements in the various categorical variables (sex, HCM status, A31P genotype, and presence or absence of LVOTO) were compared using Mann–Whitney U test or Kruskal–Wallis test with Dunn’s post-hoc comparisons. Correlations between the highest NT-proBNP values in the repeated measurement with continuous variables including age and body weight were assessed using the Spearman correlation method. Simple and multiple regression analysis after standard assumptions for linear regression were performed. Backward and forward selection techniques were performed by sequentially deleting or adding the variables with *P* < 0.15 for multiple regression analysis.

Logistic regression analysis was performed to determine the odds ratio of characteristic variables (age, sex, body weight, and A31P genotype) of developing disease. The odd ratios of developing HCM was also obtained by multiple regression analysis by including all variables used for the simple regression analysis.

### Whole-genome sequencing and variant detection

Whole-blood or tissue samples were collected for 15 research colony cats following protocols approved by the University of California Davis IACUC. Of the 15 cats, three cats had HCM but were wildtype for the A31P mutation. An additional five cats had severe or early HCM and were either heterozygous (three cats) or homozygous (two cats) for the A31P mutation. Seven cats were healthy and were wildtype for the A31P mutation. DNA was extracted from EDTA whole blood or frozen tissue samples using a commercially available kit following the manufacturers protocol (Qiagen Puregene, Germantown MD).

Ten DNA samples were submitted for WGS (McDonnell Genome Institute, Washington University, Saint Louis, MO). To ensure DNA integrity and sufficient concentration, DNA samples were run on a 2% electrophoresis agarose gel and Qubit Fluorometer (ThermoFisher, Waltham MA) assessments were performed. DNA samples that passed quality control were selected, a 450 bp KAPA PCR-free library was constructed, and 150 bp pair-ended sequencing was performed on an Illumina NovaSeq S4 instrument (Illumina, San Diego, CA). The additional five cat samples in this study previously underwent WGS using 350 bp and 550 bp PCR-free Illumina TruSeq libraries and sequenced on the Illumina HiSeq2500 sequencing platform as described^56^. All cats were sequenced to ~ 30X coverage. The generated sequences were processed as previously described^56^.

Variant call files were uploaded and analyzed via SNP & Variation Suite (SVS) commercial software (Golden Helix, Bozeman MT). Variants that had a call rate less than 80% were removed from the analysis. The remaining variants were filtered with the Ensembl FelCat Genes 94 track using SVS software; intergenic variants were removed from further analysis. A total of 142 genes were analyzed which included genes that were previously associated with HCM, suspected to be associated with HCM, as well as electrophysiology genes (e.g., genes encoding membrane transport proteins). To identify statistically significant variants, a Chi-square test was completed. Variants located in exons or in the 3’ and 5’ untranslated regions (UTR) that had a *P*-value less than 0.05 were further analyzed for the following effects: 5’—UTR premature start codon variants, disruptive inframe indels, frameshift variants, initiator codon variants, missense variants, splice acceptor/donor variants, splice region variants, and stop gain/loss variants. Variants were further filtered based on Ensembl IMPACT rating^60^. Variants that were modifiers or had a high, moderate, or low predicted effect on protein structure based on IMPACT rating and, when applicable, a SIFT (Sorting Intolerant From Tolerant) score less than or equal to 0.05 were further analyzed in a large cohort (n = 180) of unphenotyped whole-genome sequenced cats (http://felinegenetics.missouri.edu/99lives). The allele frequency of statistically significant variants was calculated for the initial 15 cats and the additional 180 cat population consisting of 43 different breeds.

## Supplementary Information

Supplementary Information.

## Data Availability

Restrictions apply to the availability of these data. Data are available from the corresponding author upon request.
