# Supplementary material for: Hypertrophic cardiomyopathy in purpose-bred cats with the A31P mutation in cardiac myosin binding protein-C
Source: Sci Rep. 2023 Jun 26;13:10319. doi: 10.1038/s41598-023-36932-5 (PMC10293195; doi:10.1038/s41598-023-36932-5)
Supplement: Supplementary file 1 — Supplementary Information. [file 41598_2023_36932_MOESM1_ESM.docx]

Supplemental Material

**Hypertrophic Cardiomyopathy in Purpose-Bred Cats with the A31P Mutation in Cardiac Myosin Binding Protein-C3**

Joshua A. Stern, DVM, PhD; Victor N. Rivas, MS; Joanna L. Kaplan, DVM; Yu Ueda, DVM, PhD; Maureen S. Oldach, DVM; Eric S. Ontiveros, PhD; Kristina B. Kooiker, PhD; Sabine J. van Dijk, PhD; Samantha P. Harris, PhD

**Supplemental Tables**

**Table S1: Median (IQR) of echocardiographic parameters are compared among cats by A31P genotype.**

| **Parameter** | **Genotype** | | | | **P-value** | | | |  |  |
| --- | --- | --- | --- | --- | --- | --- | --- | --- | --- | --- |
|  | HO (n =22) | HT (15) | WT (7) | | Overall | HO vs. HT | HO vs. WT | | HT vs. WT |  |
| IVS diastole (cm) | 0.6 (0.53 - 0.66) | 0.59 0.53-0.62) | | 0.58 (0.47 - 0.64) | 0.51 |  |  |  | | |
| IVS systole (cm) | 0.92 (0.84-1.1) | 0.97 (0.84 - 1.10) | | 0.96 (0.81 - 0.98) | 0.67 |  |  |  | | |
| LV diastole (cm) | 1.04 (0.9-1.26) | 1.11 (1.27-1.04) | | 1.45 (1.31-1.53) | 0.29 |  |  |  | | |
| LV systole (cm) | 0.36 (0.26-0.44) | 0.38 (0.35-0.5) | | 0.39 (0.34-0.45) | 0.25 |  |  |  | | |
| LVPW diastole (cm) | 0.6 (0.52 - 0.68) | 0.56 (0.51 - 0.6) | | 0.57 (0.46 - 0.58) | 0.26 |  |  |  | | |
| LVPW systole (cm) | 0.9 (0.81-1.05) | 0.93 (0.85-0.98) | | 0.84 (0.74 - 0.93) | 0.38 |  |  |  | | |
| FS (%) | 47.6 (44.3-54.5) | 49.1 (40-53.78) | | 41.9 (33.3-52.4) | 0.32 |  |  |  | | |
| LA diastole (cm) | 1.34 (1.25-1.44) | 1.36 (1.18-1.41) | | 1.25 (1.07 - 1.31) | 0.084 |  |  |  | | |
| Ao systole (cm) | 0.87 (0.85-0.94) | 0.96 (0.89-1.05) | | 0.94 (0.87-1.01) | 0.039* | 0.069 | 0.23 | > 0.999 | | |
| LA/Ao | 1.61 (1.56 - 1.67) | 1.47 (1.36-1.54) | | 1.39 (1.26 - 1.51) | 0.0002* | 0.0084* | 0.0012* | 0.8 | | |
| E Vmax (m/sec) | 0.53 (0.45-0.62) | 0.55 (0.48-0.6) | | 0.49 (0.36 - 0.61) | 0.61 |  |  |  | | |
| A Vmax (m/sec) | 0.89 (0.82 - 0.99) | 0.84 (0.74-1.01) | | 0.83 (0.76 - 0.95) | 0.7 |  |  |  | | |
| E/A (m/sec) | 0.67 (0.57-0.76) | 0.69 (0.67 - 0.79) | | 0.67 (0.54 - 0.76) | 0.5 |  |  |  | | |
| LAA Flow (m/sec) | 33.8 (30.44 - 38.17) | 36.08 (31.26-46.19) | | 29.83 (25.01 - 35.98) | 0.12 |  |  |  | | |
| Septal Bulge (mm) | 6.3 (6.1 - 7.3) | 6.33 (5.65 - 6.7) | | 5.6 (5.5 - 7.2) | 0.17 |  |  |  | | |
| Ao Vmax (m/sec) | 2.02 (1.32-3.03) | 1.98 (0.96-2.58) | | 1.4 (1 - 3.28) | 0.82 |  |  |  | | |
| P Vmax (m/sec) | 1.23 (1.06 - 1.42) | 1.09 (0.97-1.6) | | 1.88 (1.04 - 2.4) | 0.12 |  |  |  | | |

**Abbreviations:** Ao: aorta, Ao V_max_: peak velocity of aortic flow, A V_max_: peak velocity of late transmitral flow, E V_max_: peak velocity of early diastolic transmitral flow, FS: fractional shortening, HT: heterozygous, HO: homozygous, IVS: interventricular septum, LAA: left auricular appendage, LV: left ventricle, LVPW: left ventricular posterior wall, P V_max_: peak velocity of pulmonary flow. ^*^*P*<0.05

**Table S2**: **Odds ratios calculated using multivariate regression analysis.**

| **HCM status** | **Odds ratio** | **95% CI** | **P-value** |  |
| --- | --- | --- | --- | --- |
| A31P genotype: HT (vs. WT) | | 3.47 | 0.45-27.13 | 0.24 |
| A31P genotype: HO (vs. WT) | 11.8 | 1.43 – 97.14 | 0.022^*^ |  |
| Age | 0.96 | 0.90 - 1.02 | 0.18 |  |
| Sex: Female (vs. male) | 1.2 | 0.23 – 6.26 | 0.83 |  |
| Weight | 1.48 | 0.93 - 2.38 | 0.049^*^ |  |
|  |  |  |  |  |

The odds ratios in developing HCM was obtained using multiple logistic regression analysis between HCM status and A31P genotypes, age, sex, and weight.

**Abbreviations:** HT: heterozygous, WT: wildtype, HO: homozygous, CI: confidence interval. ^*^*P*<0.05.

**Table S3: Variants identified via WGS.**

| **Gene** | **No. of Variants** | **1** | **2** | **3** | **4** | **5** | **6** | **7** | **8** | **9** | **10** | **11** | **12** | **13** | **14** | **15** | **16** | **17** |
| --- | --- | --- | --- | --- | --- | --- | --- | --- | --- | --- | --- | --- | --- | --- | --- | --- | --- | --- |
| A2ML1 | 2817 | 21 |  | 2 |  |  |  |  |  |  | 2671 | 46 | 2 | 1 | 17 |  |  | 57 |
| ABCC9 | 3212 |  |  |  |  |  |  |  |  |  | 3130 | 7 | 1 | 1 | 12 |  |  | 61 |
| ACADVL | 135 | 3 |  |  |  |  |  |  |  |  | 84 | 11 |  |  | 5 |  |  | 32 |
| ACTC1 | 265 | 62 |  |  |  |  | 5 | 1 | 1 |  | 116 | 12 |  |  | 2 |  |  | 66 |
| ACTN2 | 1777 | 10 |  | 7 |  |  |  |  |  |  | 1707 | 7 |  |  | 3 |  |  | 43 |
| AGL | 2793 | 138 | 1 | 10 |  |  |  |  | 1 |  | 2482 | 38 |  |  | 11 |  |  | 112 |
| AKAP9 | 2339 |  |  |  |  |  | 7 |  | 1 |  | 2112 | 96 |  |  | 6 | 1 |  | 116 |
| ANK2 | 20645 |  |  |  |  |  | 3 |  | 2 |  | 20352 | 94 |  |  | 19 | 1 | 1 | 173 |
| ANKRD1 | 381 | 89 | 1 | 3 |  |  |  |  |  |  | 277 | 2 |  |  | 1 |  |  | 8 |
| BAG3 | 1004 | 194 |  | 15 |  |  |  |  |  |  | 731 | 23 |  |  |  |  |  | 41 |
| BRAF | 2732 |  |  | 3 |  |  |  |  |  |  | 2703 | 4 |  |  | 1 |  |  | 21 |
| CACNA1C | 5621 |  |  |  |  |  | 1 | 2 |  |  | 5486 | 11 |  |  | 11 | 1 |  | 109 |
| CACNA2D1 | 9892 |  |  | 8 |  |  | 6 |  |  |  | 9832 | 12 | 1 |  | 6 |  |  | 27 |
| CACNB2 | 17404 | 208 | 2 | 7 |  |  | 2 |  |  |  | 17106 | 26 |  |  | 5 |  |  | 48 |
| CALM2 | 196 | 2 | 1 | 16 |  |  |  |  |  |  | 174 |  |  |  | 1 |  |  | 2 |
| CALM3 | 578 | 125 | 1 | 30 |  |  | 1 |  |  |  | 411 |  |  | 1 | 3 |  |  | 6 |
| CALR3 | 792 | 6 |  | 4 |  |  |  |  |  |  | 765 | 5 |  |  | 2 |  |  | 10 |
| CASQ2 | 2368 | 32 |  | 4 |  |  |  | 1 |  |  | 2317 | 1 |  |  | 1 |  |  | 12 |
| CAV3 | 276 | 17 |  | 13 |  |  |  |  |  |  | 238 |  |  |  | 1 |  |  | 7 |
| CBL | 3009 |  |  | 4 |  |  |  |  | 1 |  | 2952 | 4 |  |  | 5 |  |  | 43 |
| PLN | 7 |  |  |  |  |  |  |  |  |  |  | 1 |  |  |  |  |  | 6 |
| CHRM2 | 54 |  |  |  |  |  |  |  |  |  | 1 | 4 |  |  |  |  |  | 49 |
| CPT2 | 396 | 2 |  | 1 |  |  | 9 | 1 |  |  | 275 | 37 |  |  | 1 |  |  | 70 |
| CRYAB | 131 | 28 |  | 1 |  |  |  |  |  |  | 93 | 3 |  |  |  |  |  | 6 |
| CSRP3 | 838 | 128 |  | 3 |  |  |  |  |  |  | 680 | 4 |  |  | 5 |  |  | 18 |
| CTF1 | 54 | 11 |  | 1 |  |  |  |  |  |  | 38 | 2 |  |  |  |  |  | 2 |
| CTNNA3 | 55759 |  |  |  |  |  |  |  |  |  | 55685 | 10 | 1 |  | 1 |  |  | 62 |
| DES | 1513 | 48 |  | 30 |  |  |  | 1 |  |  | 1365 | 11 |  |  | 12 |  |  | 46 |
| DNAJC19 | 133 | 61 |  | 3 |  |  |  |  |  |  | 66 | 2 |  |  |  |  |  | 1 |
| DOLK | 90 |  |  |  |  |  | 1 |  |  |  |  | 14 |  |  |  |  |  | 75 |
| DSC2 | 2211 | 40 |  | 18 |  |  | 3 | 1 | 1 |  | 2030 | 45 |  |  | 11 |  |  | 62 |
| DSG2 | 958 | 59 |  | 2 |  | 1 | 9 | 2 |  |  | 815 | 24 |  |  | 3 | 1 |  | 42 |
| DSP | 1274 |  |  |  |  |  | 2 | 3 |  |  | 980 | 36 | 1 |  | 9 |  |  | 243 |
| DTNA | 4355 | 19 |  | 15 |  |  |  |  |  |  | 4274 | 3 |  |  | 13 |  |  | 31 |
| ELAC2 | 1287 |  |  |  |  |  | 1 |  | 1 |  | 1204 | 28 |  | 1 | 13 |  |  | 39 |
| EMD | 38 | 3 |  |  |  |  |  |  |  |  | 29 |  |  |  | 5 |  |  | 1 |
| EYA4 | 15516 | 4 |  | 9 | 1 |  | 6 | 1 | 2 |  | 15461 | 9 |  |  | 4 |  |  | 19 |
| FHL1 | 1424 | 21 |  | 5 |  |  |  |  |  |  | 1377 | 4 |  |  | 4 |  |  | 13 |
| FHL2 | 3025 | 18 |  | 28 |  |  | 2 |  |  | 2 | 2951 | 6 |  |  | 2 |  |  | 16 |
| FKRP | 87 |  |  |  |  |  | 1 |  |  |  |  | 18 |  |  |  |  |  | 68 |
| FKTN | 2125 |  |  |  |  |  |  |  |  |  | 2109 | 6 |  |  | 2 |  |  | 8 |
| FLNC | 829 |  |  |  |  |  | 4 |  |  |  | 550 | 24 |  | 1 | 31 |  |  | 219 |
| GAA | 912 | 27 |  | 1 |  |  |  | 2 |  |  | 733 | 61 |  | 1 | 8 |  |  | 79 |
| GATA4 | 2132 | 79 |  | 23 |  |  |  |  |  |  | 1998 | 4 |  |  | 1 |  |  | 27 |
| GATA6 | 585 | 13 |  | 3 |  |  | 2 |  |  |  | 531 | 9 |  |  | 2 |  |  | 25 |
| GATAD1 | 142 |  |  |  |  |  |  |  |  |  | 136 | 1 | 1 |  |  |  |  | 4 |
| GJA5 | 43 |  |  |  |  |  | 5 |  |  |  |  | 9 |  |  |  |  |  | 29 |
| GLA | 269 | 88 | 1 | 1 |  |  |  |  |  |  | 157 | 8 |  |  | 2 |  |  | 12 |
| GPD1L | 1978 | 80 |  | 2 |  |  |  |  |  |  | 1853 | 3 |  |  | 3 |  |  | 37 |
| HCN4 | 841 | 3 |  | 5 |  |  | 1 | 1 |  |  | 717 | 11 |  |  | 1 |  |  | 102 |
| HRAS | 57 | 6 |  | 2 |  |  |  |  |  |  | 29 |  |  |  | 1 |  |  | 19 |
| ILK | 123 |  |  |  |  |  | 7 | 2 |  |  | 81 | 10 | 3 | 4 | 7 |  |  | 9 |
| JPH2 | 2513 | 76 | 2 | 19 |  |  | 3 |  | 1 |  | 2359 | 12 |  |  |  |  |  | 41 |
| JUP | 590 | 4 |  |  |  |  |  |  |  |  | 498 | 5 |  |  | 4 |  |  | 79 |
| KCNA1 | 34 |  |  |  |  |  |  |  |  |  |  | 2 |  |  |  |  |  | 32 |
| KCNA5 | 86 |  |  |  |  |  | 3 |  |  |  |  | 15 |  |  |  |  |  | 68 |
| KCND3 | 7897 | 41 |  | 2 |  |  | 2 |  |  |  | 7775 |  |  |  | 4 |  |  | 73 |
| KCNE1 | 17 |  |  |  |  |  |  |  |  |  |  | 7 |  |  |  |  |  | 10 |
| KCNE2 | 3 |  |  |  |  |  |  |  |  |  |  | 1 |  |  |  |  |  | 2 |
| KCNE3 | 4 |  |  |  |  |  |  |  |  |  |  | 1 |  |  |  |  |  | 3 |
| KCNE5 | 20 |  |  |  |  |  | 2 |  |  |  |  | 5 |  |  |  |  |  | 13 |
| KCNH2 | 542 | 3 |  | 2 |  |  | 3 | 1 |  |  | 467 | 9 |  |  | 4 |  |  | 53 |
| KCNJ2 | 353 | 158 |  | 5 |  |  |  |  |  |  | 126 | 1 |  |  |  |  |  | 63 |
| KCNJ5 | 1126 | 106 |  | 30 |  |  |  |  |  |  | 915 | 7 |  |  |  |  |  | 68 |
| KCNJ8 | 210 | 13 |  | 17 |  |  |  |  |  |  | 122 | 1 |  |  |  |  |  | 57 |
| KCNK3 | 1275 | 60 |  | 1 |  |  | 1 |  | 1 |  | 1181 | 10 |  |  |  |  |  | 21 |
| KCNQ1 | 9274 | 25 |  |  |  |  |  |  |  |  | 9227 | 3 |  |  | 7 |  |  | 12 |
| KCNQ2 | 940 |  |  |  |  |  |  |  |  |  | 844 | 12 |  |  | 3 |  |  | 81 |
| KCNQ3 | 8565 |  |  |  |  |  | 3 |  |  |  | 8482 | 14 |  |  | 3 |  |  | 63 |
| KCNT1 | 3043 | 57 |  | 1 |  |  |  | 1 | 1 |  | 2877 | 22 |  | 1 | 12 |  |  | 71 |
| KRAS | 913 | 41 |  | 2 |  |  |  |  |  |  | 862 | 4 |  |  | 0 |  |  | 4 |
| LAMA4 | 5866 | 4 |  | 5 |  |  |  |  |  |  | 5730 | 29 |  |  | 15 |  |  | 83 |
| LAMP2 | 897 | 95 |  | 3 |  |  | 4 | 2 |  |  | 761 | 13 |  |  | 4 |  |  | 15 |
| LDB3 | 2019 | 71 | 2 | 26 |  |  | 1 |  |  |  | 1828 | 17 |  |  | 6 |  |  | 68 |
| LMNA | 1017 | 30 |  | 9 |  |  | 2 |  |  |  | 900 | 7 |  |  | 11 | 1 |  | 57 |
| LRRC10 | 56 |  |  |  |  |  | 2 |  | 2 |  |  | 13 |  |  |  |  |  | 39 |
| MAP2K1 | 1500 |  |  | 49 |  |  |  |  |  | 1 | 1427 | 2 |  |  | 3 |  |  | 18 |
| MED12 | 755 |  |  | 4 |  |  | 6 | 5 | 1 | 1 | 531 | 18 |  |  | 26 | 1 |  | 162 |
| MTO1 | 720 | 13 |  |  |  |  | 4 |  |  |  | 603 | 26 | 1 |  | 8 | 1 |  | 64 |
| MYBPC3 | 694 |  |  |  |  |  | 1 |  | 1 |  | 512 | 37 |  | 2 | 30 |  |  | 111 |
| MYH6 | 874 | 2 |  |  |  |  | 2 |  | 1 |  | 711 | 16 |  |  | 20 |  |  | 122 |
| MYH7 | 909 | 5 |  | 1 |  |  | 1 |  |  |  | 669 | 36 |  |  | 26 |  | 2 | 169 |
| MYL2 | 304 | 13 |  |  |  |  |  |  |  |  | 281 | 1 |  |  | 1 |  |  | 8 |
| MYL3 | 219 | 8 |  | 1 |  |  | 3 |  |  |  | 192 | 1 |  |  | 1 |  |  | 13 |
| MYL4 | 806 | 2 |  | 2 |  |  |  |  |  |  | 786 | 1 |  |  | 3 |  |  | 12 |
| MYLK2 | 568 | 26 |  | 1 |  |  |  |  |  |  | 477 | 17 |  |  | 4 |  |  | 43 |
| MYO6 | 1939 |  |  |  |  |  | 3 |  | 1 |  | 1901 | 4 |  |  | 8 |  |  | 22 |
| MYOM1 | 2042 |  |  |  |  |  |  |  |  |  | 1962 | 19 | 2 |  | 8 |  |  | 51 |
| MYOZ2 | 1393 |  |  |  |  |  | 1 |  |  |  | 1368 | 10 | 1 |  | 1 |  |  | 12 |
| MYPN | 3407 | 30 | 1 | 4 |  |  |  |  |  | 1 | 3246 | 48 |  | 1 | 1 |  |  | 75 |
| NEBL | 17897 | 12 |  |  |  |  | 2 |  |  | 1 | 17809 | 25 |  |  | 6 | 1 |  | 41 |
| NEXN | 792 | 14 |  |  |  |  |  | 1 |  |  | 760 | 5 |  |  | 2 |  |  | 10 |
| NF1 | 4994 | 64 |  |  |  |  | 5 |  |  |  | 4648 | 57 |  | 1 | 12 | 2 |  | 205 |
| NKX2-5 | 141 | 5 |  | 6 | 1 | 1 | 9 | 2 |  |  | 48 | 20 |  | 1 | 1 | 4 |  | 43 |
| NPPA | 522 | 9 |  | 4 |  |  | 1 |  |  |  | 487 | 10 |  |  |  |  | 1 | 10 |
| NRAS | 264 | 65 |  | 1 |  |  |  |  |  |  | 182 | 1 |  |  | 4 |  |  | 11 |
| PCDH19 | 3897 | 3 |  |  |  |  | 2 |  |  |  | 3700 | 23 | 1 |  | 2 |  |  | 166 |
| PDLIM3 | 838 | 10 |  | 5 |  |  |  |  |  |  | 800 | 6 |  |  | 1 |  |  | 16 |
| PKP2 | 3435 |  |  |  |  |  | 1 |  |  |  | 3396 | 13 | 1 |  | 2 |  |  | 22 |
| PLEKHM2 | 1673 | 39 |  | 16 | 1 |  | 12 | 1 | 4 |  | 1508 | 33 | 1 | 1 | 8 |  |  | 49 |
| PRDM16 | 15971 |  |  | 12 |  | 1 | 6 |  |  |  | 15695 | 90 |  |  | 11 |  |  | 156 |
| PRKAG2 | 8730 |  |  |  |  |  |  |  |  |  | 8678 | 12 |  |  | 6 |  |  | 34 |
| PTPN11 | 3316 | 132 |  | 2 |  | 1 |  |  |  |  | 3132 |  |  |  | 6 |  |  | 43 |
| RAF1 | 561 | 41 |  |  |  |  | 1 |  |  |  | 436 | 7 |  |  | 3 | 1 |  | 72 |
| RANGRF | 111 |  | 3 | 58 |  |  |  |  |  |  | 21 | 17 | 1 |  | 1 |  |  | 10 |
| RASA1 | 2900 | 12 |  | 1 |  |  |  |  |  |  | 2834 | 5 |  |  | 7 | 1 |  | 40 |
| RBM20 | 6699 | 97 |  | 4 |  |  | 3 |  | 3 |  | 6409 | 73 |  |  | 5 | 1 |  | 104 |
| RIT1 | 124 |  |  | 8 |  |  |  |  |  |  | 109 |  |  |  | 2 |  |  | 5 |
| RRAS | 128 | 8 |  | 1 |  |  | 4 |  | 1 |  | 104 | 3 |  |  |  |  |  | 7 |
| SCN10A | 2109 |  |  |  |  |  | 10 |  |  |  | 1911 | 55 |  |  | 5 | 2 |  | 126 |
| SCN1A | 2649 | 17 |  | 4 |  |  | 1 | 1 | 1 |  | 2456 | 28 |  |  | 7 | 1 |  | 133 |
| SCN1B | 328 | 29 |  | 3 |  |  | 1 |  | 2 |  | 271 | 4 |  |  | 3 |  |  | 15 |
| SCN2B | 537 | 149 |  | 2 |  |  |  |  |  |  | 342 | 10 |  |  | 2 |  |  | 32 |
| SCN3B | 1294 | 286 | 2 | 32 |  |  |  |  |  |  | 954 | 2 |  |  |  |  |  | 18 |
| SCN4B | 1121 | 168 |  | 25 |  |  |  |  |  |  | 902 | 5 |  |  | 1 |  |  | 20 |
| SCN5A | 2296 |  |  |  |  |  | 3 | 1 |  |  | 2110 | 37 |  |  | 6 |  |  | 139 |
| SCN8A | 6497 | 129 |  | 6 |  |  | 6 | 1 |  |  | 6165 | 11 |  | 2 | 9 |  |  | 168 |
| SCN9A | 2003 | 40 | 2 | 3 |  |  | 6 | 1 | 1 |  | 1781 | 32 |  |  | 6 | 1 |  | 130 |
| SDHA | 1048 | 23 |  | 1 | 1 |  | 2 |  | 1 |  | 852 | 44 | 1 | 1 | 6 | 1 |  | 115 |
| SGCD | 6866 |  |  | 5 |  |  |  |  |  |  | 6852 |  |  |  | 2 |  |  | 7 |
| SHOC2 | 1627 | 13 |  | 5 |  | 1 | 1 |  | 1 |  | 1591 | 1 |  |  | 2 |  |  | 12 |
| SLC22A5 | 318 | 8 |  |  |  |  |  |  |  |  | 292 | 9 |  |  | 1 |  |  | 8 |
| SLC2A1 | 971 | 9 |  | 4 |  |  |  |  |  |  | 886 | 5 |  |  | 5 |  |  | 62 |
| SLMAP | 4629 | 83 |  | 21 |  |  |  |  |  |  | 4482 | 2 |  |  | 7 |  |  | 34 |
| SNTA1 | 769 | 6 | 1 | 11 |  |  |  |  |  |  | 722 | 9 |  |  | 1 |  |  | 19 |
| SOS1 | 5089 | 84 |  | 1 |  |  | 2 |  |  |  | 4933 | 5 |  |  | 8 |  |  | 56 |
| SOS2 | 3216 |  |  |  |  |  |  |  |  |  | 3126 | 18 | 2 |  | 7 |  |  | 63 |
| SPRED1 | 5465 |  |  | 3 |  |  |  |  |  |  | 5449 | 4 |  |  | 1 |  |  | 8 |
| TAZ | 79 | 2 |  | 1 |  |  |  |  |  |  | 64 | 2 |  |  | 2 |  |  | 8 |
| TCAP | 34 | 13 |  | 2 |  |  |  |  |  |  | 4 | 4 |  |  |  |  |  | 11 |
| TGFB3 | 802 | 135 |  |  |  |  |  |  |  |  | 627 | 4 |  |  | 4 |  |  | 32 |
| TMEM43 | 332 | 100 |  |  |  | 1 | 2 |  |  |  | 178 | 17 |  |  | 2 |  |  | 32 |
| TMPO | 816 | 54 |  | 1 |  |  | 4 |  | 1 |  | 657 | 58 |  |  | 1 |  |  | 40 |
| TNNC1 | 108 | 7 |  | 2 |  |  |  |  |  |  | 89 |  |  |  | 1 |  |  | 9 |
| TNNI3 | 128 |  |  |  |  |  |  |  |  |  | 121 |  |  |  | 1 |  |  | 6 |
| TNNT2 | 632 |  |  |  |  |  |  |  |  |  | 605 | 5 |  |  | 4 | 1 |  | 17 |
| TPM1 | 553 | 77 |  |  |  |  |  |  |  |  | 461 | 2 |  |  | 3 |  |  | 10 |
| TRPM4 | 2209 | 14 |  | 32 |  |  | 3 |  | 1 |  | 2015 | 42 |  |  | 14 |  |  | 88 |
| TTN | 8572 |  |  |  |  | 1 | 51 | 6 | 4 |  | 5427 | 802 |  | 1 | 65 | 5 |  | 2210 |
| TTR | 126 | 2 | 1 | 1 |  |  | 1 |  |  |  | 118 | 2 |  |  |  |  |  | 1 |
| TXNRD2 | 663 |  |  |  |  |  | 1 | 1 |  |  | 629 | 12 |  |  | 2 |  |  | 18 |
| VCL | 3004 |  |  |  |  |  |  |  |  |  | 2934 | 9 |  |  | 6 |  |  | 55 |

Annotated variants identified in eight HCM-affected and seven control cats whole genome sequenced at ~30x coverage in known and suspected HCM and electrophysiology genes. Intergenic variants were excluded from this analysis. Column headers correspond to the following:

1. 3’- UTR variant
2. 5’ - UTR premature start codon variant
3. 5’ – UTR variant
4. Disruptive inframe deletion
5. Disruptive inframe insertion
6. Frameshift variant
7. Inframe deletion
8. Inframe insertion
9. Initiator codon variant
10. Intron variant
11. Missense variant
12. Splice acceptor variant
13. Splice donor variant
14. Splice region variant
15. Stop gained variant
16. Stop lost variant
17. Synonymous variant

**Table S4.** **Statistically significant variants identified via WGS.**

| Marker | Ref | Alt | Gene | Impact | Minor Allele Freq | Major Allele Freq | *P*-value | Affected Cats | | | Control Cats | | | Cat Population | | |
| --- | --- | --- | --- | --- | --- | --- | --- | --- | --- | --- | --- | --- | --- | --- | --- | --- |
|  |  |  |  |  |  |  |  | WT | HT | HO | WT | HT | HO | WT | HT | HO |
| A2:16290308-SNV | G | A | MYL3 | MODIFIER | 0.13 | 0.87 | 0.02 | 8 | 0 | 0 | 3 | 4 | 0 | 141 | 32 | 7 |
| A2:21044679-SNV | C | A | TNNC1 | MODIFIER | 0.12 | 0.88 | 0.02 | 8 | 0 | 0 | 3 | 4 | 0 | 145 | 28 | 7 |
| B2:71164090-Del | T | - | MYO6 | MODIFIER | 0.18 | 0.82 | 0.02 | 0 | 6 | 1 | 4 | 2 | 0 | 11 | 33 | 136 |
| C1:11471173-SNV | T | C | PLEKHM2 | LOW | 0.43 | 0.57 | 0.01 | 0 | 1 | 7 | 1 | 5 | 1 | 72 | 72 | 36 |
| C1:11476559-SNV | T | G | PLEKHM2 | MODERATE | 0.40 | 0.60 | 0.04 | 5 | 3 | 0 | 1 | 4 | 2 | 32 | 73 | 74 |
| C1:11476568-SNV | G | A | PLEKHM2 | MODERATE | 0.39 | 0.61 | 0.04 | 5 | 3 | 0 | 1 | 4 | 2 | 31 | 72 | 77 |
| C1:11477761-SNV | C | T | PLEKHM2 | MODIFIER | 0.42 | 0.58 | 0.01 | 7 | 1 | 0 | 1 | 5 | 1 | 64 | 76 | 40 |
| C1:11477999-Ins | - | T | PLEKHM2 | MODIFIER | 0.29 | 0.71 | 0.04 | 1 | 3 | 4 | 3 | 4 | 0 | 96 | 70 | 14 |
| C1:157820696-SNV | T | A | SCN9A | MODIFIER | 0.12 | 0.88 | 0.03 | 1 | 6 | 1 | 5 | 2 | 0 | 147 | 31 | 2 |
| C1:169203269-SNV | T | C | TTN | MODERATE | 0.07 | 0.93 | 0.04 | 7 | 0 | 0 | 3 | 3 | 0 | 157 | 23 | 0 |
| C1:169233898-SNV-2 | C | T | TTN | MODERATE | 0.05 | 0.95 | 0.03 | 8 | 0 | 0 | 3 | 3 | 0 | 161 | 18 | 0 |
| C1:169267519-Del | T | - | TTN | LOW | 0.28 | 0.72 | 0.02 | 6 | 2 | 0 | 1 | 2 | 3 | 103 | 54 | 23 |
| C1:169413377-Del | AAGAA | - | TTN | LOW | 0.08 | 0.92 | 0.02 | 2 | 6 | 0 | 6 | 1 | 0 | 158 | 20 | 2 |
| D1:101334572-SNV | G | T | MYBPC3 | LOW | 0.27 | 0.73 | 0.04 | 0 | 3 | 5 | 2 | 4 | 1 | 109 | 56 | 15 |
| **D1:101340772-SNV** | **C** | **G** | **MYBPC3** | **MODERATE** | **0.02** | **0.98** | **0.02** | **3** | **3** | **2** | **7** | **0** | **0** | **180** | **0** | **0** |

Statistically significant variants identified in known and suspected HCM and electrophysiology genes in eight HCM-affected and seven control whole genome sequenced cats at ~30x coverage. The major and minor allele frequency is calculated for the 15 cats with known cardiac phenotype and a large cohort of 180 cats with unknown cardiac phenotype. Intronic and intergenic variants were excluded from this analysis. Table headers correspond to the following:

1. Ref = Reference allele
2. Alt = Alternate allele
3. Impact = Polyphen predicted functional effect
4. WT = Wildtype genotype
5. HT = Heterozygous genotype
6. HO = Homozygous genotype
